# Motion-Based Technology for People With Dementia Training at Home: Three-Phase Pilot Study Assessing Feasibility and Efficacy

**DOI:** 10.2196/19495

**Published:** 2020-08-26

**Authors:** Jindong Ding Petersen, Eva Ladekjær Larsen, Karen la Cour, Cecilie von Bülow, Malene Skouboe, Jeanette Reffstrup Christensen, Frans Boch Waldorff

**Affiliations:** 1 Research Unit for General Practice Department of Public Health University of Southern Denmark Odense Denmark; 2 Diagnostic Centre, University Research Clinic for Innovative Patient Pathways, Silkeborg Regional Hospital, Department of Clinical Medicine, Aarhus University Aarhus Denmark; 3 REHPA, The Danish Knowledge Center for Rehabilitation and Palliative Care, Odense University Hospital & University of Southern Denmark Odense Denmark; 4 The Parker Institute, Copenhagen University Hospital, Bispebjerg-Frederiksberg Hospital Copenhagen Denmark; 5 The Research Initiative for Activity Studies and Occupational Therapy, Research Unit of General Practice, Department of Public Health, University of Southern Denmark Odense Denmark; 6 Dementia Knowledge Center, Esbjerg Municipality Esbjerg Denmark; 7 Section of General Practice and Research Unit for General Practice, Department of Public Health, University of Copenhagen Copenhagen Denmark

**Keywords:** dementia, motion-based technology, virtual reality, telerehabilitation, physical training, physical and mental function

## Abstract

**Background:**

Persons with dementia tend to be vulnerable to mobility challenges and hence face a greater risk of fall and subsequent fractures, morbidity, and mortality. Motion-based technologies (MBTs), also called sensor-based technologies or virtual reality, have the potential for assisting physical exercise and training as a part of a disease management and rehabilitation program, but little is known about its' use for people with dementia.

**Objective:**

The purpose of this pilot study was to investigate the feasibility and efficacy of MBT physical training at home for people with dementia.

**Methods:**

A 3-phase pilot study: (1) baseline start-up, (2) 15 weeks of group training at a local care center twice a week, and (3) 12 weeks of group training reduced to once a week, supplemented with individual MBT training twice a week at home. A total of 26 people with dementia from a municipality in Southern Denmark were eligible and agreed to participate in this study. Three withdrew from the study, leaving 23 participants for the final analysis. Feasibility was measured by the percentage of participants who trained with MBT at home, and their completion rate of total scheduled MBT sessions. Efficacy was evaluated by physical function, measured by Sit-to-Stand (STS), Timed-Up-and-Go (TUG), 6-minute Walk Test (6MW), and 10-meter Dual-task Walking Test (10MDW); cognitive function was measured by Mini-Mental State Examination (MMSE) and Neuropsychiatric Inventory-Questionnaire (NPI-Q); and European Quality of Life 5 dimensions questionnaire (EQOL5) was used for measuring quality of life. Descriptive statistics were applied accordingly. Wilcoxon signed-rank and rank-sum tests were applied to explore significant differences within and between the groups.

**Results:**

As much as 12 of 23 participants (52%) used the supplemental MBT training at home. Among them, 6 (50%) completed 75% or more scheduled sessions, 3 completed 25% or less, and 3 completed between 25% and 75% of scheduled sessions. For physical and cognitive function tests, supplementing with MBT training at home showed a tendency of overall stabilization of scores among the group of participants who actively trained with MBT; especially, the 10MDW test even showed a significant improvement from 9.2 to 7.1 seconds (*P*=.03). We found no positive effect on EQOL5 tests.

**Conclusions:**

More than half of the study population with dementia used MBT training at home, and among them, half had an overall high adherence to the home training activity. Physical function tended to remain stable or even improved among high-adherence MBT individuals. We conclude that MBT training at home may be feasible for some individuals with dementia. Further research is warranted.

## Introduction

Dementia is characterized by cognitive impairments that gradually change the individual’s behavior, personality, and physical functioning [1]. As the disease progresses, individuals with dementia experience increasing difficulties with everyday tasks and often require support from caregivers to complete daily activities [2]. Worldwide, approximately 50 million people live with dementia [3], and this number will increase dramatically over the coming decades with the aging population. This will place heavy health and economic burdens on individuals, families, and society at large [1].

Many rehabilitation interventions to improve everyday life for people with dementia and their families have been explored, and many more are under consideration from research to implementation in the future [4]. Among such interventions, a suite of new technologies has gained popularity in recent years as an aid to support elderly care. Some of these technologies, such as electronic health (eHealth) and smart home devices, seem promising and feasible for the elderly both in institutions and at home [5-7], although one may be more effective than another depending on chronic diseases under management [8].

Alongside new technology development, usage of new technologies for assisting physical exercise and training as a part of disease management and rehabilitation has grown in parallel with the decentralization of health care, shifting from skilled facility care to in-home care [9,10]. Among such technologies are motion-based technologies (MBTs), also called sensor-based technologies or virtual reality. This kind of assistive technology has been approved as beneficial for stroke recovery and atraumatic brain injury rehabilitation [11,12].

For dementia, a growing body of research reports on the possibility of improving the lives of people with dementia using MBTs, but most training programs researched thus far have featured games and leisure activities and were conducted in group settings [13]. MBTs for individuals with dementia training at home and focusing on physical and cognitive function through structured exercise have rarely been studied, although many people with early stage dementia are capable of using computers and touchscreen tablets [14]. A single-case feasibility study from Canada testing a 2-week virtual reality training for a patient with dementia reported that an exercise-based virtual reality intervention was tolerated well by the patient; however, the patient’s balance and mobility remained unaffected [15].

Persons with dementia tend to be vulnerable to mobility challenges and hence face a greater risk of fall and subsequent fractures, morbidity, and mortality [16]. Physical exercise involving the movement of skeletal muscles has been associated with improvement of balance, functions, and mood among older people, aside from other health improvement benefits [17,18]. Therefore, strengthening body capacity levels through exercise among people with dementia may not only reduce these risks but also has the potential to improve physical and cognitive function as well as overall well-being [19-22], although some benefits of exercise are still debated [23].

The current public policy in Denmark aims to promote living at home for as long as possible, providing the elderly with in-home care and support, including those with dementia [24]. Many individuals with early stage dementia can live independently and tend to do so; approximately 60% of all persons with dementia live in their own home [25]. Effective home-based rehabilitation using MBTs is desirable as compared with center-based rehabilitation, an intervention which has demonstrated efficacy but presents challenges for individuals who require assistance to attend in-person visits [26]. A combination of home- and center-based training could present flexibility for people with dementia and their carers, serving to stabilize physical and cognitive function without losing social contacts.

However, it is unknown how individuals with dementia living at home respond to this type of intervention, or its potential benefits. Prior to implementing and evaluating the effects of MBTs in a larger randomized controlled trial (RCT), it is relevant to understand whether individuals will accept and implement MBTs, as well as the completion rate and preliminary physical and cognitive effect outcomes. We thus conducted this pilot study aiming to test the feasibility and efficacy of MBT training at home for people with dementia.

## Methods

### MBT Hardware and Software

The MBT used for this project utilized an online administration system developed by Welfare Denmark (Wellfaster), which has been tested in people who were at higher risk of fall in Denmark [27].

**Figure 1:**
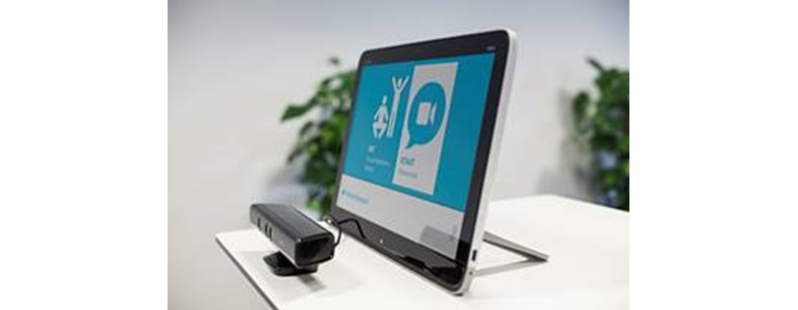
Model layout of the motion-based technology for persons with dementia training at home (Welfare Denmark [Wellfaster]).

The MBT hardware consisted of a touchscreen, a Microsoft Kinect camera, and a modem (Figure 1). The screen was placed in a room with at least 1.5 × 3 m of space to perform the exercises, and with no sharp light, which would disable registration of the participant’s movements by the camera.

The MBT training program software consisted of 142 exercises, covering all major muscle groups. The exercise program was integrated with a calendar system, allowing a physiotherapist to schedule and select daily training exercises to fit each individual participant’s needs.

**Figure 2:**
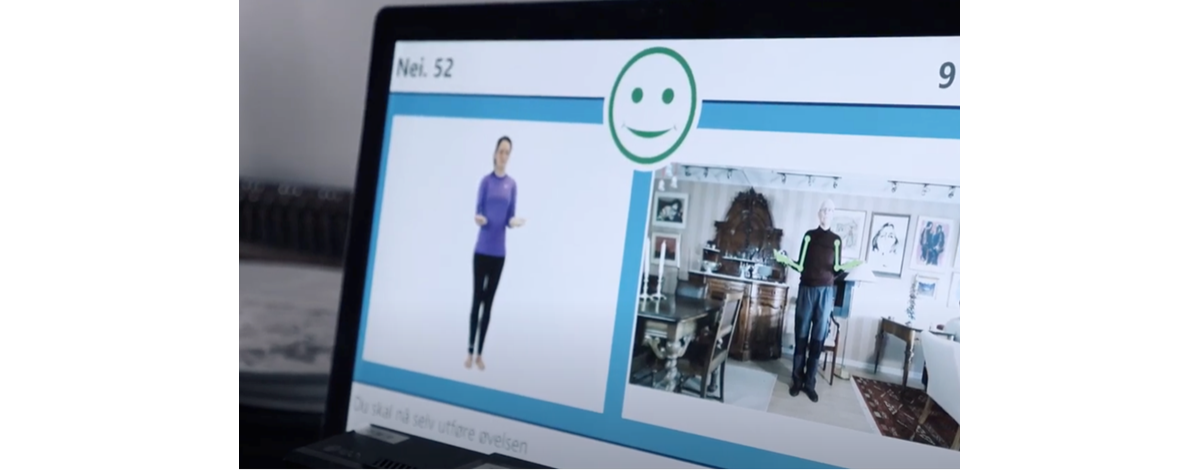
Model layout of movement detection and visual feedback with a green smiling icon (Welfare Denmark [Wellfaster]).

The system, when initiated, guided the participant through various exercises via text, recorded instructions, and animations. The Kinect camera detected the individual’s movements and corrected possible errors with onscreen feedback; once the participant successfully completed each exercise, visual feedback in the form of a green smiling icon was displayed onscreen. If the exercise was properly performed, the green smiling icon appears on the screen; if performed incorrectly, then a frowning icon appears (Figure 2). After finishing the daily training session, the participants received visual feedback displaying the percentage of correctly completed exercises.

The training data were transmitted to a physiotherapist in the form of graphic charts including the date of the training performed, percentage of correctly completed exercises, and number of training sessions completed that day. If the participants did not train as agreed or trained but with discrepancies in terms of quality, an email was automatically generated to the responsible physiotherapist to follow-up with necessary measures.

### Study Participants

The study participants were from Esbjerg, a municipality located in southwestern Denmark with approximately 115,000 inhabitants. The municipality offers rehabilitation to citizens with dementia, comprising physical training and social and psychological support at local care centers free of charge. Support is also offered to relatives caring for individuals with dementia [28].

For this pilot study, participant selection proceeded through 2 steps: (1) once a person was diagnosed with dementia at a memory clinic in Esbjerg in 2016-2017, s/he was asked whether s/he was willing to be contacted by the municipality’s Dementia Knowledge Center (DKC); (2) among those who consented to further contact, a nurse from the DKC contacted and used the inclusion criteria to invite potential participants to a start-up meeting at the center.

The nurse at the DKC selected the study participants based on a diagnosis of mild to moderate dementia from the municipality memory clinics. Other inclusion criteria included participants (1) with a Mini-Mental State Examination (MMSE) score ≥18 diagnosed from the memory clinics, (2) over 50 years of age, (3) living at home, (4) sufficient fluency in Danish to participate in tests and interviews, and (5) a close relative in daily contact with the participant who also possessed sufficient fluency in Danish to participate.

In addition, we excluded participants who (1) lived at a nursing home, (2) had a diagnosis of other serious physical or psychiatric illness, including severe sight or hearing disabilities that could affect their ability to participate, or (3) were participating in other intervention projects or drug trials.

### Pilot Intervention

**Figure 3:**
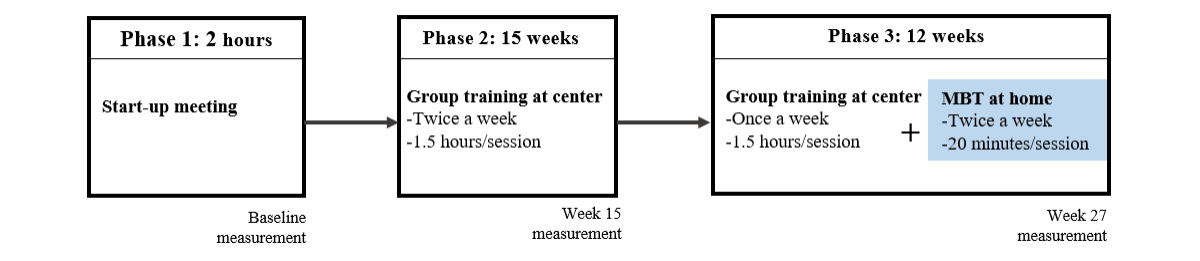
Pilot study phases.

This study intervention had 3 phases, illustrated in Figure 3.

Phase 1, which was conducted in 2016 and 2017, consisted of a 2-hour start-up meeting held at the DKC. During the meeting, a physiotherapist verbally introduced the detailed study plan to the participant and the caregiver. Practical operation of the MBT device, communications from the screen to the participant, and feedback methods were introduced. The individuals who agreed to participate in the study provided their consent for participation (regardless of which training option was self-chosen) and completed the baseline questionnaire survey and further physical and cognitive function measurements. Those who agreed to train supplementing with MBT received a follow-up home visit for MBT device installation, by either a technical support person or the physiotherapist, if the participant had difficulties with unfamiliar persons.

Phase 2 consisted of 15 weeks of group physical training at the local care center in 2016-2017 for all the study participants; physical training took place twice a week for 1.5 hours per session. A physiotherapist from the local care center facilitated group physical trainings, comprising exercises for balance, coordination, strength, and cardio adjusted to participants’ strength, flexibility, and endurance. While the individuals completed the physical training session, caregivers were provided with a separate room with free coffee, facilitating an opportunity to meet and talk to other caregivers.

Phase 3 was a 12-week training including the MBT intervention. In this phase, the participants, based on the decisions they made in Phase 1, self-directed to either (1) continue with the group training at the local training center twice a week for 1.5 hours per session, or (2) continue with the group training at the local training center but reduced to once a week for 1.5 hours per session, supplemented with individual MBT training at home twice a week for 20 minutes each session.

### Variable Measurements

The study outcomes were feasibility and efficacy. We defined the feasibility as the percentage of the participants who trained with MBT at home, examining their completion rate for the scheduled total 24 sessions over the 12 weeks of the intervention. We defined the efficacy as improvement of participants’ physical and cognitive function and quality of life (QoL).

### Baseline Demographic Characteristics of Participants and Caregivers

The characteristics of participants at baseline were obtained using semistructured questionnaires administered by a project nurse during the start-up meeting, gathering information on sociodemographics, dementia diagnosis, chronic diseases, and social relations. The baseline characteristics of caregivers were also self-reported in the questionnaire and included sociodemographic information and relationship to the participant.

### Physical Function Measurements

To evaluate the effects of physical training, including MBT, we measured participants’ physical functions at baseline, at Week 15, and at Week 27 using 4 measurements discussed next, all of which are common physical performance measures for muscular strength, balance, walking ability, and gait speed in older adults, including those with a dementia diagnosis [29-31].

#### Sit-to-Stand Test

The Sit-to-Stand (STS) test determines a person’s functional level by quantifying performance of lower extremity muscles [32]. STS is performed using a chair without armrests, with a seat height of 43 cm; participants are required to stand up from the chair and then sit back. The number of repetitions completed (sit to stand) over 30 seconds was counted. The same chair was used for all participants during all 3 study phases.

#### Timed-Up-and-Go Test

The Timed-Up-and-Go (TUG) test evaluates dynamic balance and is used to assess persons at risk of falling due to gait problems and balance. It is a reliable and validated test in the elderly population to measure functional ability and clinical changes over time [29]. TUG measures the time in seconds that participants require to rise from a straight-backed chair without using their arms, walk 3 meters, turn around, walk back to the chair, and sit down. The same chair was used in the TUG test for all participants for all 3 study phases.

#### 6-Minute Walk Test

The 6-minute Walk Test (6MW) was originally developed for patients with heart failure [33], and it is a reliable and validated measure in evaluating walking ability among older people with dementia [30]. The participants were asked to walk for 6 minutes as far as they could, at their usual pace. The distance in meters they walked in 6 minutes was recorded. The participants could stop and/or rest if they felt it necessary.

#### 10-Meter Dual-Task Walking Test

Previous studies have shown that dual-task training improves dual-task performance in people with mild to moderate dementia [34,35]. The 10-meter Dual-Task Walking Test (10MDW) required participants to walk for 10 m at a comfortable, normal pace without assistance or mobility aids (straight walk) and then to walk the same distance back (turn walk/dual task). The participants were asked to wear comfortable footwear to the test. The time to traverse 10 m on the two occasions was also averaged to calculate their gait velocity.

### Cognitive Function and Quality of Life Measurements

#### Mini-Mental State Examination

Even if the participants had fulfilled the MMSE score inclusion criteria, there was a time gap from the date of diagnosis by the local memory clinic to the date of study entrance, and changes in cognitive functions vary among the study participants. We, therefore, again measured their cognitive function by using MMSE at the baseline and at Week 27 (the end of the supplemental MBT), administered by a consultant specialized in dementia. MMSE is a validated and widely used screening tool for assessing cognitive impairment and tracking cognitive changes over time. It briefly measures several domains, including orientation to time and plan, immediate recall, short-term verbal, memory, calculation, language, and construct ability [36]. MMSE has in total 30 points, and in DKC daily practice, a score of 25-30 is considered mild dementia, a score of 18-24 is considered moderate dementia, and any score under 18 is categorized as severe dementia.

#### Neuropsychiatric Inventory-Questionnaire

We applied the Neuropsychiatric Inventory-Questionnaire (NPI-Q) to assess the presence of the participants’ dementia symptom severity and distress [37]. NPI-Q includes 12 categories of behavioral disturbance: delusions, hallucinations, anxiety, depression/dysphoria, agitation/aggression, elation/euphoria, disinhibition, irritability/lability, apathy/indifference, motor disturbance, nighttime behavior problems, and problems with appetite/eating. For this study, we presented a total NPI-Q score as the sum of the total severity score and total distress score [38], which were self-reported by the participant.

#### Quality of Life

Participants’ and their caregivers’ QoL at baseline, Week 15, and Week 27 were measured using the Euro Quality of Life 5D questionnaire (EQOL5), which is a short, simple, validated questionnaire for the health-related QoL assessment [39]. It contains 5 three-level dimensions covering morbidity, self-care, usual activities, pain/discomfort, and anxiety/depression. A single summary score ranging from 0 to 100 is given by the participant to indicate his/her health status. The higher the score, the better the participant’s QoL.

### Statistical Analysis

The proportion of participants who trained with supplemental MBT training at home, the successful completion rate, the correction rate (ie, the percentage of all training sessions completed correctly), and the characteristics of the participants at baseline according to whether they supplemented with MBT training at home were all described statistically. The differences between the two groups were tested using Pearson chi-squared test for the categorical variables, or two-sample *t* tests for the continuous variables.

Physical and cognitive functions and QoL on 3 occasions (Phases 1, 2, and 3) were presented as medians with first and third quartile (25%-75%). Given the relatively small number of study participants and the potential skewing of their functional data distribution, we therefore applied Wilcoxon signed-rank and rank-sum tests to explore the significant differences between 2 occasions (Phase 1 and Phase 2, and Phase 2 and Phase 3), both within and between the groups with and without supplemental MBT training at home.

Subgroup analysis was conducted among the participants according to their MBT training completion status: those who trained more actively (completion of 75% or more sessions as scheduled) and those who trained less actively (completion of 25% or less sessions as scheduled) at home using MBT.

The person who conducted the questionnaire surveys, the person who collected the MBT training data, and the person who analyzed the data were independent and blinded for each other’s tasks.

Stata Statistical Software Release 16 (StataCorp) was used for statistical analysis, and *P* values less than .05 were considered statistically significant.

### Consent and Ethical Considerations

Individuals with dementia and their relatives were instructed verbally and in writing regarding the project. Those who wished to participate in the study signed a consent statement. This study has been submitted to the University Scientific Ethics Committee and assessed as requiring no need for notification (Journal number 2016-41-4844). The Danish Data Inspectorate approved the project (Journal number 2016-41-4844).

## Results

### Selection of Participants

A total of 49 potential participants were identified with a diagnosis of dementia from Esbjerg memory clinics within 12 months prior to the study baseline. Among them, 18 were excluded as they did not fulfill the inclusion criteria, which left 31 persons eligible for study participation. Of those 31, 5 declined to participate in the study.

**Figure 4:**
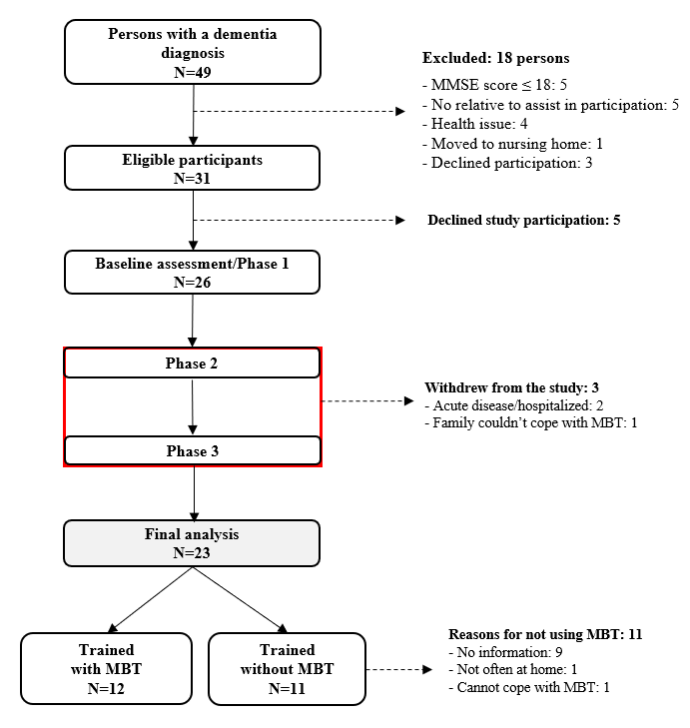
Flowchart of study participation.

A total of 26 participants were assessed during the start-up meeting (baseline) in 2016-2017. However, 3 persons withdrew from the study due to acute illness/hospitalization (n=2) or because the family could not cope with MBT technological difficulties (n=1). This left 23 participants for the final analysis, including 12 who supplemented with MBT training at home and 11 who only completed the center-based group training with no additional supplementation (Figure 4).

There were different reasons as to why the 11 participants chose not to train with MBT at home: not at home very often (n=1), had a hard time coping with MBT technology (n=1), and no information was provided (n=9).

### Characteristics of the Participants at Baseline

Table 1 presents the characteristics of the 23 participants at baseline in the 2 groups. The mean age of the 12 participants who supplemented their training with at-home MBT was 75.6 years (SD 6.4), and the mean age of those who trained without MBT was 79.1 years (SD 4.4).

Overall, the 2 groups had no significant difference in age, gender, health conditions, physical functioning, socioeconomic status, or caregiver characteristics. However, those who trained with MBT had on average a higher QoL and a slower 10MDW than those who did not train with MBT at baseline (*P*<.05).

**Table 1:** Characteristics of the participants at baseline (n=23) by the status of supplemental at-home MBT^a^ training.

Characteristics						Supplemented with MBT at home					
						MBT (n=12)		No MBT (n=11)		*P* value	
**Participants**											
	Age, mean (SD)				75.6 (6.4)			79.1 (4.4)		.14	
	**Gender**									.06	
		Male			10			5			
		Female			2			6			
	Height, cm (SD)				173.6 (7.4)			166.7 (7.2)		.04	
	Weight, kg (SD)				80.5 (13.4)			71.5 (12.9)		.12	
	**Health conditions**										
		**Dementia type**								.18	
				Alzheimer	5			3			
				Vascular dementia	1			4			
				Dementia with Lewy body	3			3			
				Mild cognitive impairment	0			1			
				Unspecified dementia	3			0			
		**Computed tomography/Magnetic resonance imaging scanning for dementia diagnosis**								.29	
				Yes	12			10			
				No	0			1			
		**Comorbidity**									
				Diabetes	2			0		.16	
				Heart disease	4			3		.75	
				Chronic obstructive pulmonary disease	1			1		.95	
				Depression	4			3		.75	
	**Cognitive and physical functioning**										
		**MMSE^b^ scores**								.008	
				Mild (25-30)	10			5			
				Moderate (18-24)	2			0			
				No report				6			
	Neuropsychiatric Inventory-Questionnaire, mean (SD)				14.3 (8.4)			11.6 (2.5)		.67	
	European Quality of Life 5 dimensions, mean (SD)				78.5 (20.1)			59.7 (19.1)		.01	
	Sit-to-Stand in numbers^c^, mean (SD)				13 (3.2)			12 (2.7)		.53	
	Timed-Up-and-Go in seconds, mean (SD)				8.4 (4.2)			7.7 (1.9)		.60	
	10-meter Dual-Task Walking Test in seconds, mean (SD)				10.5 (2.9)			8.2 (1.8)		.036	
	6-minute Walk Test in meters, mean (SD)				370.5 (156.8)			383.1 (74.7)		.81	
	**Socioeconomics**										
		**Education**								.54	
				Have education	10			8			
				Do not have any education	2			3			
		**Living alone**								.13	
				Yes	2			5			
				No	10			6			
		**House living status**								.51	
				Own home	10			9			
				Elderly community	0			1			
				Other	2			1			
**Caregivers**											
	**Living together with dementia patient**									.13	
			Yes		10			6			
			No		2			5			
	**Contact with the person with dementia**									.09	
			Daily		11			8			
			Several times per week		1			3			
	**Education**									.95	
			Have some education		11			10			
			No education		1			1			
	**Currently working**									.90	
			Yes		3			3			
			No		9			8			
	European Quality of Life 5 dimensions, mean (SD)				79.8 (20.1)			70.5 (16.5)		.24	

^a^MBT: motion-based technology.

^b^MMSE: Mini-Mental State Examination.

^c^Number of repetitions completed (sit to stand) over 30 seconds.

### Feasibility of Using MBT Training at Home

Of the 23 participants, 12 (52%) trained with MBT at home. Among them, 6 (50%) completed (≥75%) all 24 sessions as scheduled, with a correction quality (ie, the percentage of all training sessions completed correctly) ranging from 70% to 94%, 3 completed 25%-75%, and 3 completed less than 25% as scheduled. The successful intervention implementation rate among those who trained with MBT at home was thus 50% (6/12; Table 2).

**Table 2:** Completed sessions as scheduled (n=24) among 12 participants who trained with supplemental at-home motion-based technology.

Completion as scheduled	Participants, n (%)	Completed sessions, n (correction rate %)^a^
≥75% completion^b^	6 (50)	25 (90), 26 (70), 30 (84), 32 (93), 50 (74), 62 (94)
25%-75% completion	3 (25)	9 (92), 10 (85), 16 (82)
≤25% as completion	3 (25)	1 (57), 2 (42), 3 (58)

^a^The percentage of all training sessions that participants completed correctly.

^b^All participants in this group completed all training sessions as scheduled.

### Development of Physical and Cognitive Functions and Quality of Life

Table 3 lists the physical and cognitive function as well as QoL measured at each occasion among the participants who trained with and without MBT at home.

In general, physical function measured using the 10MDW among the participants who trained with MBT showed a tendency of improvement throughout the study, including during the period of MBT implementation from Week 15 (9.1 seconds) to Week 27 (8.0 seconds), whereas other measures for physical function including STS, TUG, and 6MW, showed a tendency to stabilize in both groups.

No significant change was observed in MMSE or NPI-Q throughout the study between the 2 groups. Regarding QoL, the participants showed a decline from Week 15 to Week 27 in both groups during the period of MBT implementation. However, their caregivers showed two tendencies for this period: a declining tendency of QoL score among those who trained with MBT, and an increasing tendency of QoL score among those who trained without.

A Wilcoxon signed-rank test showed no statistically significant differences between the groups at Week 27 as well as within the group between Week 15 and Week 27 for all measurements (ie, the period when MBT was implemented).

**Table 3:** Physical and mental function on 3 occasions among the participants who trained with (n=12) and without (n=11) MBT^a^ at home.

Functional status			Median (interquartile range Q1-Q3)					
			P1: Baseline		P2: Week 15		P3: Week 27	
**Physical function**								
	**Sit-to-Stand in numbers^b^**							
		With MBT		14 (10-16)		14 (12-16)		14 (12-16)
		Without MBT		12 (10-14)		12 (11-15)		13 (11-16)
	**Timed-Up-and-Go in seconds**							
		With MBT		6.7 (6.7-8.5)		6.7 (5.5-7.9)		6.5 (5.5-7.8)
		Without MBT		8.0 (6.1-9.2)		7.1 (5.7-9.1)		7.8 (5.9-8.5)
	**10-meter Dual-Task Walking Test in seconds**							
		With MBT		10.0 (9.2-12.2)^c^		9.1 (7.5-9.8)^d^		8.0 (7.1-8.9)
		Without MBT		8.6 (7.2-9.5)		8.7 (7.5-9.5)		8.6 (7.2-10.1)
	**6-minute Walking Test in meters**							
		With MBT		443 (345-474)		416 (350-472)		418 (334-501)
		Without MBT		385 (311-457)		339 (304-468)		360 (334-493)
**Cognitive function and quality of life**								
	**MMSE^e^ score**							
		With MBT		26 (24-27)				24 (22-28)
		Without MBT		24 (24-26)				24 (23-26)
	**NPI-Q^f^ score**							
		With MBT		10 (3-17)		7 (2-12)		10 (3-12)
		Without MBT		10 (4-11)		13 (5-23)		12 (8-12)
	**QoL^g^ score**							
		With MBT		80 (72-80)^c^		85 (70-90)		78 (68-80)
		Without MBT		60 (50-75)		75 (70-80)^d^		70 (60-80)
	**Caregivers’ QoL score**							
		With MBT		90 (70-92)		94 (80-100)^d^		83 (67-98)
		Without MBT		70 (50-90)		78 (75-83)		90 (70-90)

^a^MBT: motion-based technology.

^b^Number of repetitions completed (sit to stand) over 30 seconds

^c^Significant difference between two groups at baseline (*P*<.05)

^d^Significant difference within the group between baseline and Week 15 (*P*<.05)

^e^MMSE: Mini-Mental State Examination.

^f^NPI-Q: Neuropsychiatric Inventory-Questionnaire.

^g^QoL: quality of life.

### Subgroup of Participants Who Trained Actively With MBT

A subgroup analysis showed that among those who more actively trained with MBT at home (completion rate ≥75% as scheduled) had a significant improvement of 2.1 seconds in the 10MDW test, from 9.2 at Week 15 to 7.1 seconds at Week 27 (*P*=.031), as shown in Table 4. The other 3 tests (STS, TUG, and 6MW), however, showed a tendency of stabilization in this period. For the group of participants who trained less actively with MBT (completion rate ≤25% as scheduled), all 4 physical function tests showed a tendency of either no change or a decline from Week 15 to Week 27.

MMSE and NPI-Q seemed stable among those who more actively trained with MBT. QoL of the participants and their caregivers both had a tendency of decline regardless of whether they trained more or trained less actively with MBT. Again, except 10MDW, all other test measurements were statistically insignificant between or within the group between Week 15 and Week 27.

**Table 4:** Physical and mental function of participants who more actively (n=6) and less actively (n=3) used MBT^a^ at home.

Functional status				Median (interquartile range Q1-Q3)				
				P1: Baseline		P2: Week 15		P3: Week 27
			
**Physical function**								
	**Sit-to-Stand in numbers^b^**							
		More active	13 (11-14)		14 (12-15)		14 (12-15)	
		Less active	16 (9-17)		12 (11-20)		11 (10-16)	
	**Timed-Up-and-Go in seconds**							
		More active	7.1 (6.7-9.6)		6.6 (5.5-8.5)		6.5 (5.4-7.8)	
		Less active	6.7 (6.6-6.7)		6.4 (5.4-6.9)		6.5 (5.4-7.8)	
	**10-meter Dual-Task Walking Test in seconds**							
		More active	10.1 (9.7-14.5)		9.2 (7.8-9.7)		7.1 (6.4-7.9)^c^	
		Less active	10.0 (8.8-10.0)		7.8 (7.2-9.5)		8.5 (8.4-12.2)	
	**6-minute Walking Test in meters**							
		More active	443 (348-455)		423 (370-480)		424 (345-548)	
		Less active	345 (345-462)		400 (330-464)		334 (277-465)	
**Cognitive function and quality of life**								
	**MMSE^d^ score**							
		More active	25 (23-27)				24 (20-25)	
		Less active	25 (25-26)				22 (22-28)	
	**NPI-Q^e^ score**							
		More active	6 (1-12)		8 (3-11)		6 (5-11)	
		Less active	15 (2-20)		7 (1-17)		10 (0-24)	
	**QoL^f^ score**							
		More active	80 (76-80)		90 (80-90)		80 (70-100)	
		Less active	70 (60-75)^g^		70 (60-90)		65 (60-75)	
	**Caregivers’ QoL score**							
		More active	78 (65-90)		86 (74-94)		71 (61-78)	
		Less active	90 (45-100)		100 (40-100)		95 (60-100)	

^a^MBT: motion-based technology.

^b^Number of repetitions completed (sit to stand) over 30 seconds.

^c^Among those who more actively and less actively trained with MBT between Week 15 and Week 27, Wilcoxon signed-rank test *P*=.031.

^d^MMSE: Mini-Mental State Examination.

^e^NPI-Q: Neuropsychiatric Inventory-Questionnaire.

^f^QoL: quality of life.

^g^Among those who more actively and less actively trained with MBT at home at baseline, rank-sum test *P*=.048.

## Discussion

### Principal Findings

This pilot study tested whether supplementing with MBT training at home is feasible and can improve physical and cognitive function as well as QoL of people with mild dementia. Among the 23 participants in the final analysis, more than half (12/23, 52%) trained with MBT and among that group, half (6/12, 50%) had an overall high adherence to this home training activity. Supplementing with MBT training at home showed a tendency to stabilize physical and cognitive functioning; physical function even improved in some participants who more actively trained with MBT. However, our results revealed a tendency of declining QoL with MBT implementation, for both the participants and their caregivers, a finding which requires further study.

Studies of physical training using MBT at home (with a similar platform) in people with dementia are sparse, and it is therefore difficult to compare our study with others conducted under similar circumstances. A literature review of 45 studies concludes that people with dementia are able to independently use touchscreen technology for cognitive rehabilitation with featured games or leisure activities [40]. Other researchers report that although people with early stage dementia are largely not confident with new technology devices, half were able to use a tablet computer independently [14]. In our study, we also found half of the participants had high adherence with the use of MBT at home, which shows consistency with the aforementioned studies in a broader sense.

Learning a new technology can be both physically and emotionally challenging for elderly individuals, especially for those with dementia. These challenges can include unfamiliarity with new technology operation, lack of understanding the purpose, fear of mistakes, or forgetfulness about how the technology works, which can affect the participants’ adherence to using MBT training at home [41]. Adaptability to new technology is lower among the elderly than among younger populations [42]. For those with cognitive impairment, adaptability can be even lower. Indeed, cognitive impairment of persons with dementia can limit memory, attention, concentration, visuality, execution, and speed, which can affect both performance and the experience of using MBT. Additionally, the introduction to MBT can be critical, influencing the patients and their caregivers’ choice or desire to learn to use MBT [41], even if the program, including the MBT, was free of charge and offered by the municipality.

Alongside the intervention, the QoL of the participants and their caregivers, among those who trained with MBT, showed a tendency of decline. This has been interviewed with 4 pilot study participants (2 with dementia and 2 caregivers) which revealed that caregivers enjoyed the center-based training because it presented an opportunity to talk to other caregivers while the group training took place; MBT training at home was viewed as less enjoyable because it was difficult to adapt to the new technology. MBT provides many small exercises for training different body muscles; although an introduction and demonstration of MBT was provided prior to the intervention, elderly persons and especially those with dementia may benefit from a longer learning process to become comfortable with new technology. There is an ongoing study evaluating the participants’ experiences of using MBT and their interactions with the physiotherapist during the training process, which may potentially provide valuable information for further refinement of this intervention.

Compared with the group training at the local center, the individual home training environment reduces social contacts, social activity, and social coherence. Social participation is important for well-being and maintenance of cognitive function [43,44]. However, a previous study of people with Alzheimer’s dementia living at home showed that fewer than 40% had participated in social activities outside their home more than once a week, and nearly 30% never left their home for social activities [45]. While technological interventions have the potential to improve care, some may also create barriers to social participation, resulting in overall dissatisfaction and other adverse health consequences for both patients and their family caregivers [46].

Family caregivers play an important role in the everyday lives of individuals with dementia. For people with dementia living at home, based on evidence from the United States, approximately 75% of the care for these individuals is provided by family and friends [47]. In this study, we do not know how many participants needed and/or received support/assistance from caregivers, or how much, while training with MBT at home. A study found that people with dementia who lived with their partners tended to rely on them for support when implementing a new electronic assistive device [48]. In this pilot study, it was also often up to relatives whether to activate the MBT during the start-up meeting. Caring for a person with dementia is stressful in daily life. Too much demand for assistance with MBT training at home may have caused distress to the relative/caregiver, thereby reducing their own QoL, and additional stress if they are also unfamiliar with the MBT program’s setup and operation.

Teaching and training the caregivers on hands-on skills with the assistive device may increase their ability to facilitate, support, and assist the people with dementia. A randomized controlled trail of 153 community-dwelling individuals with dementia found that home-based exercise training combined with teaching behavior management techniques to caregivers improved physical health and depression, compared with only routine care [49]. In this RCT, caregivers showed their capacity to encourage and supervise exercise participation, thus increasing the patients’ physical and social activity and providing a successful integrated home-exercise program model that may apply for further research.

Despite the abovementioned, MBT training at home can provide freedom and flexibility compared with training in groups at a center. People with dementia face barriers to exercise, and researchers and health professionals are actively working together to find effective ways to deliver exercise to people with dementia; it is still unknown whether center- or home-based training is superior [50,51]. As populations age, and more elderly tend to stay at home for as long as possible, a growing trend for home-based care demands has arisen, including rehabilitation. As Denmark provides free health care to citizens, evaluation of the efficacy of such interventions should also consider the economic component.

### Study Limitations

A relatively small sample size is one of the limitations of this study, especially the subgroups, which may have floor and ceiling effect for the results’ reliability [52]. However, given that this is a pilot study to test the feasibility and efficacy of MBT training at home, a small sample size study is a rational design in terms of resource use and financial cost. Based on this pilot study’s results, a larger study with improved strategic design will be implemented, preferably as a randomized control trial.

Beyond the sample size limitation, this pilot study also has generalizability weaknesses. Only those with newly diagnosed dementia who were interested in physical exercise training were invited to the start-up meeting. During that meeting, participants were drawn only from among those who were willing to use the MBT training at home and comply with the MBT regimen. Therefore, the participants in our study cannot be viewed as representative of all individuals with dementia who might train with MBT.

Moreover, involvement of users as informants and co-designers is vital for successful rehabilitation interventions and research. A systematic review of 26 publications involving people with dementia in the development of supportive IT applications concluded that involvement in all phases lead to better applications as well as empowering effects on the users [53]. We lacked this aspect in this pilot study; although the MBT platform was intended to be easy to operate, it had never been tested in people with dementia.

Of the 12 participants who trained with MBT at home, we saw 2 polar opposite phenomena: a group of participants who actively trained with a high correction rate (ranging from 70% to 94%), and a group who trained much less with a lower correction rate (ranging from 42% to 58%). For this latter group, we lack full knowledge on their reasons for not completing the scheduled training sessions. Despite difficulties in adapting to new technology, incorrect performance during MBT training may cause emotional distress, leading to dropping out or withdrawing. However, this pilot intervention lacked a comprehensive formal evaluation to identify what factors affected MBT completion rates not limited to adaptation to the new technology, such as physical, emotional, social, and spiritual well-being perspectives.

We implemented MBT twice a week together with group training at a local center once a week. Center-based group training is part of the usual care in Denmark among this population and is continuously offered to older citizens with dementia in Esbjerg, as it has shown positive effects, including social participation. As this MBT self-training at home is a pilot study with uncertainty about its effects, the weekly group training was included to at least maintain the patients’ and their caregivers’ social contacts. However, such a combination may have influenced the outcomes to some extent. As this pilot study showed positive potential, a further study might involve a large sample RCT designing MBT as one single arm.

### Conclusion

More than half of the participants with dementia trained with MBT at home, and among them, half had an overall high adherence to the preset MBT training activity. Physical and cognitive tests showed stable scores throughout the intervention and to some extent even improvements. We conclude that MBT training at home for people with dementia may be feasible for some people with dementia together with center-based training. Further research is warranted to identify the capacities and challenges of MBT implementation and successful completion, preferably targeting both the participants and their family caregivers.

## Abbreviations

6MW: 6-minute Walk
10MDW: 10-meter Dual-Task Walking
DKC: Dementia Knowledge Center
EQOL5: European Quality of Life 5 dimensions questionnaire
MBT: motion-based technology
MMSE: Mini-Mental State Examination
NPI-Q: Neuropsychiatric Inventory-Questionnaire
QoL: quality of life
RCT: randomized controlled trial
STS: Sit-to-Stand
TUG: Timed-Up-and-Go
